# Review of Post-embedding Immunogold Methods for the Study of Neuronal Structures

**DOI:** 10.3389/fnana.2021.763427

**Published:** 2021-10-14

**Authors:** Ronald S. Petralia, Ya-Xian Wang

**Affiliations:** Advanced Imaging Core, National Institute on Deafness and Other Communication Disorders, National Institutes of Health, Bethesda, MD, United States

**Keywords:** synapse, post-embedding, pre-embedding, immunolabeling, immunoperoxidase, Lowicryl, EPON

## Abstract

The post-embedding immunogold (PI) technique for immunolabeling of neuronal tissues utilizing standard thin-section transmission electron microscopy (TEM) continues to be a prime method for understanding the functional localization of key proteins in neuronal function. Its main advantages over other immunolabeling methods for thin-section TEM are (1) fairly accurate and quantifiable localization of proteins in cells; (2) double-labeling of sections using two gold particle sizes; and (3) the ability to perform multiple labeling for different proteins by using adjacent sections. Here we first review in detail a common method for PI of neuronal tissues. This method has two major parts. First, we describe the freeze-substitution embedding method: cryoprotected tissue is frozen in liquid propane *via* plunge-freezing, and is placed in a freeze-substitution instrument in which the tissue is embedded in Lowicryl at low temperatures. We highlight important aspects of freeze-substitution embedding. Then we outline how thin sections of embedded tissue on grids are labeled with a primary antibody and a secondary gold particle-conjugated antibody, and the particular problems encountered in TEM of PI-labeled sections. In the Discussion, we compare our method both to earlier PI methods and to more recent PI methods used by other laboratories. We also compare TEM immunolabeling using PI vs. various pre-embedding immunolabeling methods, especially relating to neuronal tissue.

## Introduction

**Figure 1:**
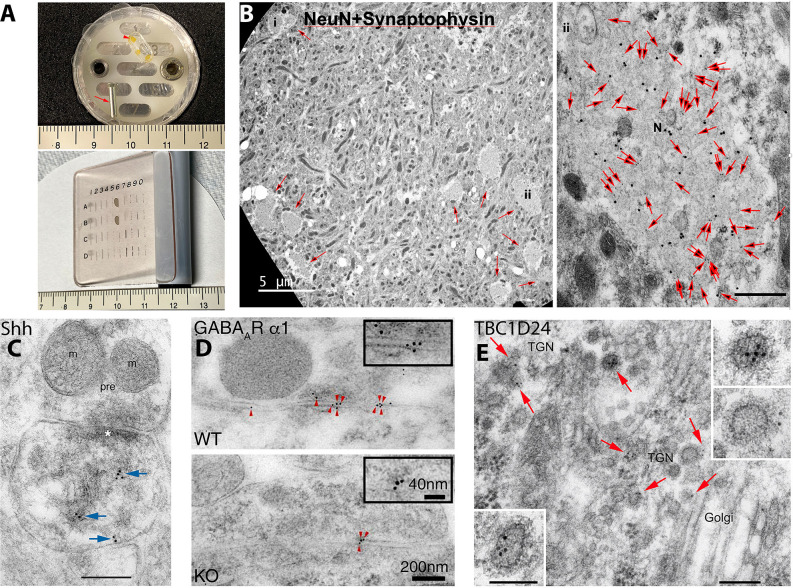
Examples of post-embedding immunogold labeling (PIL) using the protocol presented in the methods of this review. **(A)** Items used in our PIL method. The top image shows an aluminum peg (arrow) and flat-embedding mold; a sample block with three pieces of braintissue is included (arrowhead). The aldehyde-fixed andglycerol-cryoprotected tissue is placed on double-stick tape on the peg and plunged into liquid propane in a Leica CPC. Then the frozentissue is placed in the flat-embedding mold in the Leica AFS in a solution of methanol and uranyl acetate at −90°C. After infiltration with cold Lowicryl, embedding is finished with UV polymerization. Note that the embedded tissue is yellow due to the uranyl acetate. Tissue blocks are sectioned on an ultramicrotome and the thin sections placed on grids, and then immunogold-labeled while attached to a Hiraoka support plate (bottom image); note that labeling will occur on both sides of the section. See text for more details. Centimeter rulers are shown in images. **(B)** Novel clusters of large, NeuN-positive, degenerate, swollen presynaptic terminals (N; small arrows in left image) are found in the CA1 region of the hippocampus in 12-month-old mice. They are more common in DPP6-KO mice (shown); DPP6 is an auxiliary subunit of a voltage-gated potassium channel. In these swollen terminals, NeuN (20 nm gold) completely colocalizes with the presynaptic terminal protein, synaptophysin (10 nm gold; all 10 nm gold particles in themicrograph are indicated with large arrows in the right image) and labeling for both proteins is low outside of the swelling; this is consistent with light microscope observations. The location of the high magnification image (labeled #ii) is indicated in the lower right area of the low magnification image on the left. The scale bar on the right image is 500 nm. From Figure 4 of Lin et al. (2020; open access-http://creativecommons.org/licenses/by/4.0/). **(C)** PIL (10 nm gold; arrows) of sonic hedgehog (Shh) in the CA1 region of the mouse hippocampus, showing labeling associated with postsynaptic tubulovesicular structures and the spine membrane. m, mitochondrion; pre, presynaptic terminal; asterisk, postsynaptic density. The scale bar is 200 nm. From Figure 4 of Rivell et al. (2019; open access). **(D)** PIL (5 nm gold; arrowheads) of the GABA_A_ α1 receptor at inhibitory synapses in the CA1 region of the mouse hippocampus, showing a significant reduction in labeling in the Shisa7 KO compared to WT; Shisa7 is a GABA_A_ receptor auxiliary subunit protein that controls benzodiazepine actions. From Figure 2 of Han et al. (2019; AAAS). **(E)** PIL (10 nm gold; arrows) of TBC1D24 in the CA1 region of the mouse hippocampus, showing a concentration of labeling in clathrin-coated vesicles of the trans-Golgi network (TGN) in neuron somas. TBC1D24 mediates vesicle trafficking important for neuronal signal transmission, and its mutations are associated with epilepsy, deafness, and other disorders. Scale bars are 200 nm for the lower-magnification and 100 nm for the higher-magnification insets. From Figure 4 of Tona et al. (2019; Oxford University Press). Note for **(D,E)**, that AAAS and Oxford University Press, respectively, as stated in the author information, permit authors to use figures from their previously published works in subsequently published works.

Immunogold labeling for transmission electron microscopy (TEM) can be performed either before (pre-embedding immunogold; PrI) or after (post-embedding immunogold; PI) embedding (Merighi, 1992; Bendayan, 2000). The entire tissue block is committed to one labeling procedure with PrI, while embedded tissue used for PI labeling (PIL) can be sectioned many times and individual sections can be studied with different antibodies. Early PIL studies in the nervous system successfully labeled small molecules such as amino acid neurotransmitters (Ottersen, 1989; Phend et al., 1992) and some larger neurotransmitter receptors (Flucher and Daniels, 1989). Later, PIL was developed for glutamate receptors (GluRs) of the brain (Baude et al., 1993; Phend et al., 1995) and cochlear hair cell synapses (Matsubara et al., 1996). The Baude and Matsubara methods use a low-temperature-embedding media, Lowicryl, to help retain antigenicity, although Phend et al. (1995) argued that improvement in antigenicity was due to surface properties of Lowicryl rather than cold embedding. We developed our PIL method from that of (Matsubara et al., 1996; Petralia and Wenthold, 1999). Here we present the revised method (Figure 1) and compare it to other PIL methods as well as to PrI labeling.

## Our PIL Techniques

### Chemicals

1. Lowicryl HM20 (Polysciences, Warrington, PA) is a nonpolar, hydrophobic resin. We mix 2.98 g of Crosslinker D with 17.02 g of Monomer E and then add 0.1 g of Initiator C. The methanol that we use is bottled over a molecular sieve to reduce water absorption (Fluka, Ronkonkoma, NY).
2. Polyethylene glycol (20,000 MW) to reduce aggregation of gold particles: mix 0.005 g in 1 ml 1% normal goat serum (or equivalent serum) in Tris-buffered saline-Triton X-100 (TBST).
3. Sodium borohydride/glycine treatment: mix 0.01 g sodium borohydride and 0.0375 g glycine (50 mM) with 10 ml TBST.
4. TBST buffer: mix 100 ml of 0.05 M Tris, pH 7.4, with 900 ml of 0.9% NaCl and 1 g of Triton X-100.

### Instruments-CPC

1. Cryoprotected specimens are frozen in a Leica EM CPC (Leica Microsystems, Buffalo Grove, IL; no longer sold by Leica; alternative-EMS-002, EMS, Hatfield, PA) in liquid propane at −184°C cooled with liquid nitrogen.
2. Tissue is placed on double-stick tape on an aluminum stub with a fine brush or other applicator, and excess liquid can be removed using the brush.
3. Once frozen, the tissue is carried in liquid nitrogen in a small, aluminum transport unit to the Leica freeze-substitution device (AFS); alternatively, tissue can be stored in liquid nitrogen for later processing. Be cautious in handling frozen tissue and always cool instruments in liquid nitrogen before contacting the tissue on the stub.

### Instruments-AFS

1. In the AFS, we use a flat-embedding method. Three aluminum chambers each contain seven wells; the bottom of the chambers is lined with a clear plastic film to facilitate removal of the polymerized Lowicryl+tissue blocks.
2. Wells are filled with methanol + 1.5% uranyl acetate.
3. The metal stub holding the tissue on double-stick tape is placed with the tissue end in the methanol/uranyl acetate solution in the wells using forceps pre-chilled in the liquid nitrogen in the transport unit.
4. The tape with tissue is teased off the metal stub using a pre-chilled scalpel. Typically, we place two pieces of tissue-on-tape in each well. Keep the ends of instruments within the chamber at all times while manipulating the tissue and leave the nitrogen gas flow control (TF) fully-open during these procedures; this will ensure that the instruments stay cold and dry.
5. We use a 142-h sequence in the AFS, with T1 = −90°C/32 h, S1 = 4°C/h, T2 = −45°C/50 h, S2 = 5°C/h, and T3 = 0°C/40 h. Our AFS model is no longer sold, so this sequence may need to be modified in newer Leica AFS models.

### Tissue Preparation

1. Animals are cardiac-perfused with 0.12 M phosphate buffer (PB) wash and then with 4% paraformaldehyde plus 0.5% glutaraldehyde in 0.12 M PB.
2. The initial fixative should be at warm-room temperature to preserve microtubules, and the initial wash-out should be limited to ~30 s.
3. The tissue is post-fixed for 2 h at 4°C and washed 3× over an hour in 0.1 M PB with 4% glucose.
4. Brain and other organs may be cut on a Leica Microslicer (or equivalent vibratome) at 200–350 μm.
5. Tissue is cryoprotected with glycerol in 0.1 M PB (30 min each at 10%, 20%, 30%, and then overnight in 30%).
6. Tissue is plunge-frozen in a Leica CPC (described above).
7. Frozen tissue is immersed in 1.5% uranyl acetate in methanol in a Leica AFS at −90°C. Initially, in our 142-h sequence, we pause the sequence and resume in the early afternoon, to finish around noon 6 days later.
8. On the 3rd day, the AFS will be at −45°C and we begin the substitution procedure into Lowicryl. First, liquid in the chambers is exchanged with methanol 3× quickly and double-stick tape pieces are removed. Then we substitute every 2 h in 1:1 and 2:1 Lowicryl in methanol, and pure Lowicryl 2×.
9. On the 4th day, the Lowicryl is changed, and tissue pieces are positioned in the wells. The final level of Lowicryl is reduced so that it just reaches the tops of the wells; this makes it easier to remove the samples after UV polymerization, which continues from days 4–6 when the temperature goes from −45°C to 0°C.

### Immunolabeling

1. Thin sections are cut on a Leica Ultramicrotome and mounted on grids coated with Coat-quick “G” coating pens (EMS). Sections are ~100–130 nm in thickness; we use these thicker sections since Lowicryl can be fragile with TEM. Alternatively, one can cut thinner sections (~60 nm) and place them on formvar/carbon film-covered grids, but these can only be labeled from one side.
2. Grids are attached to Hiraoka support plates; these are no longer available, but reasonable substitutes are sold by EMS and other companies.
3. Grids are incubated in 0.1% sodium borohydride + 50 mM glycine in TBST for 10 min.
4. Grids are incubated in 10% normal goat serum (or other serum) in TBST for 10 min, and in primary antibody in 1% serum/TBST overnight at 4°C.
5. The remaining steps are at room temperature. Grids are washed in TBST 3× quickly and placed in TBST for 10 min.
6. Grids are washed again in TBST 3× quickly and placed in 1% serum/TBST for 10 min.
7. Grids are placed into secondary immunogold-conjugated antibodies at 1:40 in 1% serum/TBST + 0.5% polyethylene glycol for 1 h. For double-labeling (Figure 1B), the two primary antibodies are combined in one solution, as are the two secondary antibodies.
8. Grids are washed 3× in TBST and 6× in water (fast) and dried (use filter paper to remove any remaining liquid).
9. Grids are stained in 1% uranyl acetate for 5 min and washed 2–3× and dried, and then stained in 0.3% lead citrate/1% 10 N NaOH for 3 min and washed 2–3× and dried. Filter (0.22 μm) the uranyl acetate and lead citrate before use.

## Discussion

### Comparison With Other PIL Techniques

**Figure 2:**
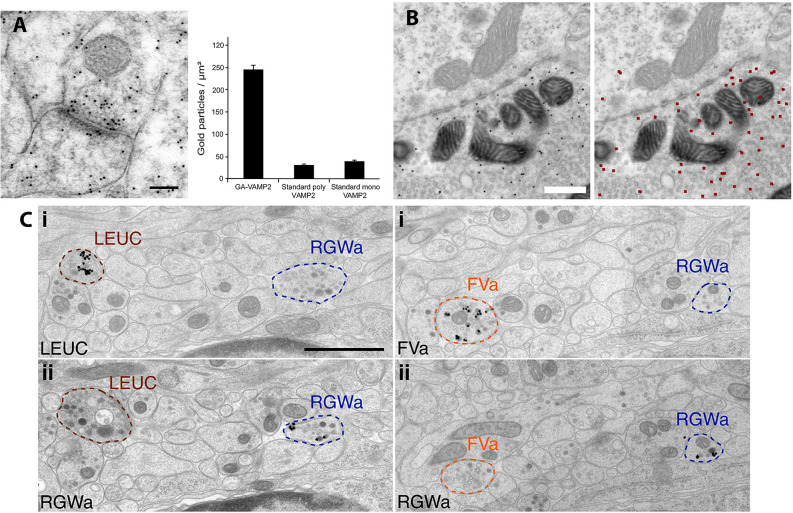
Examples of variation in PIL protocols. **(A)** PIL of the synaptic protein, VAMP2, in the CA1 region of the rat hippocampus is significantly enhanced when the antibody is raised against a glutaraldehyde-fixed antigen (polyclonal; GA-VAMP2; image shown), compared to standard polyclonal and monoclonal VAMP2 antibodies. The scale bar is 100 nm. From Figure 4 of Hussain et al. (2019; open access). **(B)** PIL for GFP in calyx synapses in the medial nucleus of the trapezoid body, following injection of an EGFP-tracer compound. Gold particles are evident in the presynaptic terminal in the left image; the right image shows the annotated output of identified gold particles (colored red), as determined by the deep learning-based software tool, “Gold Digger.” The scale bar is 500 nm. From Figure 5 ofJerez et al. (2021; open access—http://creativecommons.org/licenses/by/4.0/).**(C)** PIL labeling with neuropeptide antibodies of axons from neural tissue in the larval marine annelid worm, *Platynereis*, embedded in epon and labeled with silver-enhanced, 0.8 nm gold. Neurite-specific labeling is demonstrated in serial sections seven sections apart, using the neuropeptides, leucokinin (LEUC), RGWamide (RGWa), and FVamide (FVa). The scale bar is 1 μm. From Figure 2 of Shahidi et al. (2015; Creative commons attribution license).

Inner ear structures (e.g., cochlea) are difficult to fix adequately with cardiac perfusion; various methods of local fixative-immersion/perfusion may be used with (especially for synapses; Matsubara et al., 1996; Davies et al., 2001) or without (Andrade et al., 2012; Fang et al., 2015; also Royaux et al., 2003) cardiac perfusion. Zhang et al. (2016) review their modification of our method to study GluR distribution in rat retina, which labeled poorly for NMDA receptors compared to brain. Labeling was improved with “pH-shift fixation” in which pieces of the retina are dissected out in 4% paraformaldehyde at pH 7.4 and then placed first in 4% paraformaldehyde at pH 6.0 for 20–30 min, followed by 4% paraformaldehyde plus 0.1% glutaraldehyde at pH 10.5 for 10–20 min. Another method to improve labeling is to use smaller gold (e.g., 5 nm), which may increase labeling density (Merighi, 1992; Petralia et al., 2010). Ultra-small gold may improve labeling further, although these smaller gold particles cannot be identified with conventional TEM without employing difficult enhancement procedures (Tao-Cheng et al., 2021). Thus, one method of PIL of GluRs on grids utilized 1.4 nm gold with silver enhancement (Baude et al., 1993); also, these authors achieved even higher labeling density with the 1.4 nm gold/silver method with fixed, frozen sections on grids, without embedding media. Using these methods, they determined that the metabotropic GluR, mGluR1α, preferentially localizes to the perisynaptic zone of synapses. Hussain et al. (2019) reported that the antibodies raised to glutaraldehyde-fixed synaptic proteins improved their binding to aldehyde-fixed proteins (Figure 2A). Jones (2016) describes a method of PI of paraformaldehyde-fixed human tissue using an alternative Lowicryl, K4M. K4M has a higher freezing point than HM20 so that the lowest temperature for the substitution was −35°C. Since K4M is hydrophilic while HM20 is hydrophobic, Jones noted a problem of wetting the block face during ultramicrotomy. K4M’s hydrophilic property may help improve immunolabeling in some cases (Roth et al., 1981), e.g., triple immunogold labeling at the neuromuscular junction achieved by Flucher and Daniels (1989). The addition of water to K4M may increase labeling, but typically HM20 gives better labeling than K4M (Bendayan et al., 1987).

In addition to low-temperature Lowicryl’s, other acrylic resins (LR White, LR Gold) and epoxy resins (epon, Durcupan) have been used successfully as PI-embedding media (Timms, 1986; Bendayan et al., 1987; Paik et al., 2021; Suzuki et al., 2021). Zhang et al. (2015) and Zhang and Morales (2019) used double-immunogold labeling of LR White-embedded sections to show that two neurotransmitters are located in microdomains of the same axons. They were fixed with paraformaldehyde and picric acid with a low level of glutaraldehyde, and later steps included tannic acid and then uranyl acetate prior to embedding. Picric acid, tannic acid, and uranyl acetate have been utilized to improve ultrastructure and/or immunolabeling (Somogyi and Takagi, 1982; Phend et al., 1995; Zhang and Morales, 2019). In this study, there was definitive labeling although ultrastructure was not very good. Yamashita (2016) describes an LR White method of PI with good immunogold labeling and ultrastructure. It is a complex protocol, including two fixatives made from paraformaldehyde, calcium and magnesium chloride, and sucrose, embedding in LR White, heat-induced antigen retrieval on grids prior to primary and secondary antibody labeling, and several staining steps beginning with a post-fixation in glutaraldehyde plus tannic acid, followed by osmium tetroxide, uranyl acetate, and lead citrate.

Epoxy resins such as epon are great for ultrastructural preservation but are known to reduce the antigenicity of large proteins such as membrane receptors (Bendayan et al., 1987; Phend et al., 1995; Brorson, 1998), although they generally work well for small molecules such as neurotransmitters (Ottersen, 1989; Phend et al., 1992; Paik et al., 2021; the latter embedded in Durcupan). In fact, the high levels of glutaraldehyde that are usually included in fixatives for neurotransmitter studies actually are necessary to retain the small amino acid-type neurotransmitter molecules (Ottersen, 1989). Nevertheless, special protocols have been developed to retain the antigenicity of larger molecules in epon-embedded tissues. Zhong et al. (2013) describe a method for PI of synaptic proteins in hippocampal slice cultures embedded in epon, based on the original method developed in the 1990s (Phend et al., 1995; Valtschanoff and Weinberg, 2001). This method produces both good ultrastructure and good immunolabeling. It begins with a strong fixative containing paraformaldehyde, picric acid, and a high concentration (2.5%) of glutaraldehyde. No osmium tetroxide is used since this is known to decrease antigenicity (Roth et al., 1981; Phend et al., 1995), but subsequent steps include tannic acid, uranyl acetate, and platinum chloride, all in a maleate buffer. In contrast, the following methods do include osmium tetroxide. Jerez et al. (2021) used hot water (93–95°C) to unmask antigens (injected/traced EGFP) at synapses in Durcupan-embedded brains; notably, this study describes a learning-based software tool for autonomous gold identification (Figure 2B). Shahidi et al. (2015) describe a method of high-pressure freezing and freeze-substitution into epon, used for serial immunogold labeling of neural tissue in sections from a larval marine worm (Figure 2C). They found that, in spite of the presence of osmium and epon, short amidated neuropeptide antigens retain good antigenicity in the tissue. They were able to track immunogold labeling of 11 neuropeptide antibodies in neurites. Interestingly, they used ultra-small immunogold (0.8 nm; AURION) with silver enhancement on the grids. Grid-labeling was done on modified microwell mini-tray plates (NUNC™ brand). Kuwajima et al. (2020) studied late-phase long-term potentiation (L-LTP) with PIL of hippocampal slices fixed with glutaraldehyde/paraformaldehyde and osmium tetroxide and embedded in LX-112, a resin similar to epon. This elaborate protocol involved optogenetic stimulation of CA3 neurons in the slices, with light-inducing L-LTP in target synapses of the CA1, microwave-enhanced fixation, tyramide-signal amplification, gold enhancement (similar to silver enhancement) of immunogold, and serial EM-3D-reconstruction.

In conclusion, a number of PIL techniques can be used to obtain good immunogold labeling while retaining reasonable ultrastructure. Our method has proven successful for a variety of antigens and tissues, possibly due to simple fixation procedures, cold embedding, and the use of Lowicryl, although others have succeeded without these factors.

### Unique Advantages of PIL for Studies of Synapses and Neurites

PIL has at least three advantages over most other methods for studying synapse and neurite ultrastructure: higher resolution, the opportunity to co-label multiple proteins, and more accurate quantification (Merighi, 1992). Gold-particle location may be ≤ 21 nm to the actual antigenic site, based on the length of the double-IgG plus gold particle radius for 10 nm gold (Merighi, 1992). Matsubara et al. (1996) found that definitive labeling for a membrane-bound antigen labeled with 15 nm gold could be ≤ 28 nm from the membrane. This level of accuracy provides precise protein positioning along the width (lateral/tangential; Nusser et al., 1994; Petralia et al., 1998; Valtschanoff and Weinberg, 2001) and depth (Matsubara et al., 1996; Xiao et al., 1998; Petralia et al., 2001; Valtschanoff and Weinberg, 2001) of synapses. Nusser et al. (1994) used PIL to show that the metabotropic GluR, mGluR1α, and AMPA GluRs segregate along the width of synapses, with mGluR1α concentrated mostly perisynaptic to the postsynaptic density (PSD). Additionally, PIL was used to show that Homer, a protein binding to mGluRs peripheral to the postsynaptic membrane and IP3 receptors in the endoplasmic reticulum of the spine, is concentrated in deeper regions of the PSD (Xiao et al., 1998; Petralia et al., 2001). Valtschanoff and Weinberg (2001) used PIL to demonstrate different depths for various proteins in NMDA receptor protein complexes, with NMDA receptors close to the postsynaptic membrane, and several associated proteins in two or more zones subjacent to them. There are numerous examples of co-labeling using gold particles (Figure 1B) and comparative quantification (Figure 1D) of PIL at synapses. Flucher and Daniels (1989) used triple labeling to show two zones of labeling at neuromuscular junctions, with acetylcholine receptors and 43 kd protein at the crests of postsynaptic folds and ankyrin in the troughs of folds. Nusser et al. (1994) demonstrated with double-labeling the subsynaptic segregation of mGluR1α and an AMPA receptor at cerebellar Purkinje cell synapses. Rubio and Wenthold (1997, 1999) used single+double PIL with AMPA receptors to demonstrate that various types of GluRs are differentially distributed at apical vs. basal dendrite synapses of fusiform cells of the dorsal cochlear nucleus. Many studies have used PI to show changes in protein levels at synapses in KO animals, e.g., in hippocampal synapses, reduction of an NMDA receptor in Neto KO mice (Wyeth et al., 2014), and GABA receptors in Shisa7 KO mice (Han et al., 2019; Figure 1D).

### Comparison of Post- and Pre-embedding Immunolabeling

Here we compare PIL to pre-embedding immunolabeling including immunoperoxidase (peroxidase-catalyzed reaction product) and immunogold (Tao-Cheng et al., 2021). These pre-embedding methods are generally more sensitive and provide denser labeling than PIL because embedding media may cause some loss of antigen sensitivity (Priestley et al., 1992); however, in some circumstances, embedding may actually preserve some antigenicity (Bendayan et al., 1987). But pre-embedding immunoperoxidase and immunogold have some disadvantages compared to PIL. In pre-embedding, antibodies may have difficulties reaching all antigen sites, due to variations in membrane permeability, isolation in compartments, size of immunogold-conjugated antibodies, and dense molecular regions such as the PSD (Bendayan et al., 1987; Priestley et al., 1992; Baude et al., 1995; Bendayan, 2000; Tao-Cheng et al., 2021). For example, Baude et al. (1995) note problems in PrI labeling of some AMPA GluRs at synapses due to lack of penetration of antibodies in the PSD. Also, PrI may not be readily quantified because labeling is not as discreet as PI; usually small gold (0.8 or 1.4 nm) is used for PrI and must be silver (or gold)-enhanced, producing final particles that are larger/more variable, resulting in less-accurately-resolved antigen sites. In addition, the accumulation of the pre-embedding-derived gold may not be consistent throughout the section due to permeability problems (Tao-Cheng et al., 2021), in contrast to PIL on the section surface (Bendayan, 2000). Nevertheless, several studies have used PrI to localize proteins to subzones of synapses; thus, Baude et al. (1993) demonstrated preferential labeling of mGluR1α at the synapse periphery, and vertical movement with stimulation of PSD-associated proteins was shown for Shank (Tao-Cheng et al., 2010) and SynGAP (Yang et al., 2011). Chen et al. (2011) achieved even greater localization accuracy at the synapse using PrI; they discerned the different vertical positions of the C vs. N ends of the PSD-95 molecule, an accuracy almost as good as that obtained at synapses with PI (e.g., Valtschanoff and Weinberg, 2001). Some accuracy in the localization of proteins also has been achieved using pre-embedding immunoperoxidase labeling in subzones of axons (Zhang et al., 2015) and synaptic spines (Petralia et al., 2010; Alexander et al., 2021); the latter studies of spines improved accuracy with silver/gold enhancement.

Findings often are improved by combining PIL (e.g., more accurate localization/quantification) and pre-embedding immunolabeling (e.g., increased labeling), including several studies discussed above (Baude et al., 1993; Rubio and Wenthold, 1997; Petralia et al., 2010; Zhang et al., 2015). For example, Zhang et al. (2015) used two double-labeling protocols—pre-embedding immunoperoxidase+immunogold and quantification of double-PIL to corroborate their findings of dopaminergic+glutamatergic microdomains in axons.

## Author Contributions

RP and Y-XW wrote the section on our techniques and reviewed the manuscript. RP wrote other parts of the manuscript and prepared the figures. All authors contributed to the article and approved the submitted version.

## Conflict of Interest

The authors declare that the research was conducted in the absence of any commercial or financial relationships that could be construed as a potential conflict of interest.

## Publisher’s Note

All claims expressed in this article are solely those of the authors and do not necessarily represent those of their affiliated organizations, or those of the publisher, the editors and the reviewers. Any product that may be evaluated in this article, or claim that may be made by its manufacturer, is not guaranteed or endorsed by the publisher.
